# Homozygosity for a novel *INHA* mutation in two male siblings with hypospadias, primary hypogonadism, and high-normal testicular volume

**DOI:** 10.1530/EJE-21-1230

**Published:** 2022-03-02

**Authors:** Esra Arslan Ates, Mehmet Eltan, Bahadir Sahin, Busra Gurpinar Tosun, Tuba Seven Menevse, Bilgen Bilge Geckinli, Andy Greenfield, Serap Turan, Abdullah Bereket, Tulay Guran

**Affiliations:** 1 Marmara University Pendik Training and Research Hospital, Genetic Diseases Diagnostic Center, Istanbul, Turkey; 2 Department of Pediatric Endocrinology and Diabetes, Marmara University, School of Medicine, Istanbul, Turkey; 3 Department of Urology, Marmara University, School of Medicine, Istanbul, Turkey; 4 Mammalian Genetics Unit, MRC Harwell Institute, Oxfordshire, UK

## Abstract

**Background:**

The human *INHA* gene encodes the inhibin subunit alpha protein, which is common to both inhibin A and B. The functional importance of inhibins in male sex development, sexual function, and reproduction remain largely unknown.

**Objective:**

We report for the first time two male siblings with homozygous *INHA*mutations.

**Methods:**

The medical files were examined for clinical, biochemical, and imaging data. Genetic analysis was performed using next-generation and Sanger sequencing methods.

**Results:**

Two brothers complained of gynecomastia, testicular pain, and had a history of hypospadias. Biochemistry revealed low serum testosterone, high gonadotropin and anti-Mullerian hormone, and very low/undetectable inhibin concentrations, where available. Both patients had azoospermia in the spermiogram. We have identified a homozygous 2 bp deletion (c.208_209delAG, R70Gfs*3) variant, which leads to a truncated INHA protein in both patients, and confirmed heterozygosity in the parents. The external genital development, pubertal onset and progression, reproductive functions, serum gonadotropins, and sex hormones of mother and father, who were heterozygous carriers of the identified mutation, were normal.

**Conclusion:**

Homozygosity for *INHA* mutations causes decreased prenatal and postnatal testosterone production and infertility in males, while the heterozygous female and male carriers of *INHA* mutations do not have any abnormality in sex development and reproduction.

## Introduction

Inhibin A and B are heterodimeric proteins belonging to the transforming growth factor β superfamily, capable of suppressing pituitary follicle-stimulating hormone (FSH) secretion. Inhibin is produced mostly by the granulosa cells in the ovary and Sertoli cells in the testis (1, 2). Inhibins contain either a β_A_- or β_B_-subunit and a common α-subunit, which is encoded by *INHA* (3, 4). Activins are functional counterparts of inhibins, being dimers of the β-subunits with the ability to stimulate FSH secretion. The α-subunit binds competitively to the β-subunits to form inhibin, thereby reducing the formation of activin homodimers; but inhibin is also able to block the binding of the activins to the activin receptors, thereby inhibiting activin signalling (5). Besides the endocrine regulation of FSH biosynthesis, animal and *in vitro* cell studies have demonstrated that inhibin also has autocrine and/or paracrine actions that regulate gametogenesis and steroidogenesis (6, 7, 8, 9, 10). Knockout of *Inha* in mice results in the development of gonadal stromal tumours and cachexia-related death in both sexes (6, 7, 11, 12). Human studies have shown that certain variants and polymorphisms in coding sequence or the promoter of *INHA* are associated with primary ovarian insufficiency (POI) or primary amenorrhea in females (13). There are no reports regarding the effect of inhibins on sex development, steroidogenesis, or reproduction in males.

Here, we report for the first time two male siblings with homozygous *INHA*mutations and describe their clinical features.

## Subjects and methods

### Clinical studies

All clinical investigations and genetic analyses were performed according to the guidelines of the Declaration of Helsinki. The Ethical Committee of Marmara University, Istanbul, Turkey approved the study (09.2021.1343). Written informed consent for publication of their clinical details was obtained from the patients and the parents.

Two male siblings from a single family were evaluated for gynecomastia, hypospadias, and primary gonadal insufficiency. Detailed clinical, laboratory, and molecular characteristics of the patients and parents are described.

### DNA sequencing

Genomic DNA from peripheral blood was extracted using QIAamp DNA Blood Mini QIAcube Kit (Qiagen), according to the manufacturer’s protocols. All coding exons and exon-intron boundaries of 21 378 and 4493 genes were amplified using Twist HCE (Twist Bioscience HQ, San Francisco, CA, USA) and Clinical Exome Solution kits (SOPHiA Genetics, Boston, USA), for whole-exome sequencing (WES) and clinical exome sequencing (CES) respectively. Libraries were sequenced on the Illumina NextSeq platform (Illumina Inc., San Diego, CA, USA). Detected variation was confirmed via Sanger sequencing on an ABI Prism 3500 Genetic Analyzer (Thermo Fisher Scientific).

### Data analysis

Sophia DDM software (SOPHiA Genetics, Boston, USA) was used for data analysis. Coverage was 99.11% at a minimum depth of 25 reads for targeted regions. For variant calling, sequencing data were aligned to the human reference genome, hg19. All variants in exons and exon–intron boundaries with a variant fraction over 0.20 and having minor allele frequency (MAF) under 0.01 in GnomAD were evaluated. Genes associated with sexual development and hypogonadism (Online Mendelian Inheritance in Man), were prioritised. The Human Gene Mutation Database Professional (2020) and ClinVar databases were screened for known variants (14, 15). To evaluate the potential effects of these variants, *in silico* analyses were performed using Mutation Taster, SIFT, Provean, PolyPhen, and Combined Annotation Dependent Depletion (16, 17, 18, 19, 20). Variants were classified according to the American College of Medical Genetics (ACMG) guidelines (21).

## Results

### Case reports

#### Patient 1

**Figure 1:**
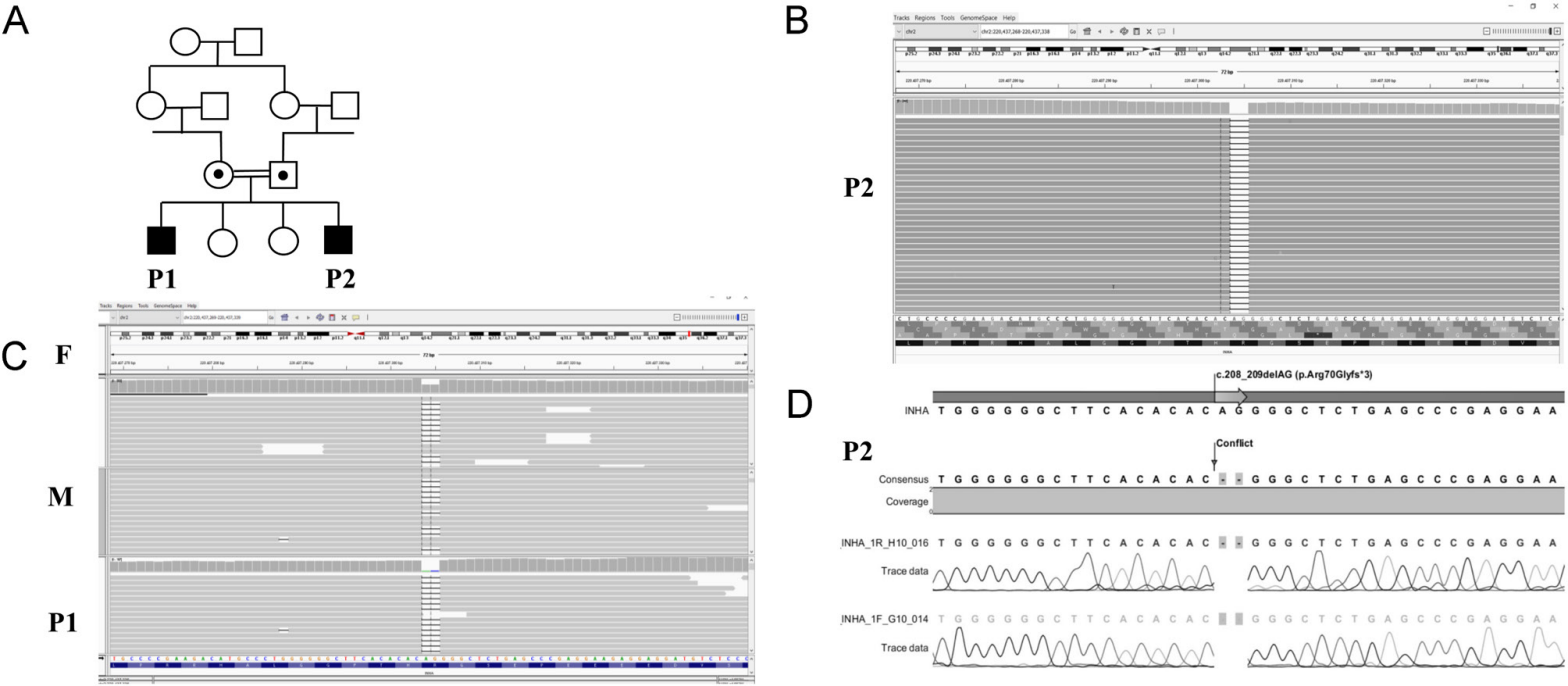
Genetic characteristics of the patients and parents with *INHA* mutations. (A) The patients (P1 and P2) were born to first-degree cousin parents. (B and C) Integrative genomics viewer images of *INHA* mutation of patients and parents*,*with a two nucleotide deletion revealed by next-generation sequencing. P1 and P2 were homozygous, father (F) and mother (M) were heterozygous carriers for the mutation. (D) Electropherogram showing the homozygous *INHA* variant in P2. A full colour version of this figure is available at https://doi.org/10.1530/EJE-21-1230.

The patient was referred for evaluation of gynecomastia at the age of 15 years and 9 months. He was born to first-degree cousin parents of Turkish descent (Fig. 1A). His past medical history was unremarkable except for a history of two operations for hypospadias, at 2 and 6 years old. At the presentation, his weight was 75.6 kg (+0.93 SDS) and his height was 163 cm (−1.42 SDS). Physical evaluation showed small, low-set, protruding ears, bilateral gynecomastia of 7 cm with Tanner stage 4 appearance. Testicular volumes were 18 cm^3^, bilaterally. He had glanular hypospadias. The penile size was normal (9 × 2.2 cm). Pubic hair was at Tanner stage 4. Systemic examinations were normal. Karyotype was 46,XY. Adrenal function tests were normal, but serum gonadotropins were elevated, suggesting hypergonadotropic hypogonadism (Table 1). Measurements of anti- Müllerian hormone (AMH) and serum inhibins in patient 1 (P1) were not possible, due to technical reasons and circumstances beyond our control. Upon follow-up, he was treated with monthly intramuscular testosterone enanthate for 4 years. At the last visit, at 23 years and5 months, his weight was 77.6 kg (+0.5 SDS) and his height was 171.3 cm (−0.84 SDS). Systemic examinations were normal, he had bilateral gynecomastia of 8 cm with Tanner stage 5 appearance. Testicular volumes were 25 cm^3^ bilaterally. After 5 years of marriage, he presented to a urology clinic due to infertility. Sperm analysis, repeated three times, revealed azoospermia. He was unavailable for further investigation.

**Table 1:** Gonadal and adrenal function test results of patients with *INHA* mutations at presentation.

	Normal range	P1	P2	Mother	Father
Age (years)		15 ^9/12^	12 ^7/12^	50	55
Karyotype		46, XY	46, XY		
FSH (mIU/mL)	1.27–19.26	41.16	74	2.98	1.18
LH (mIU/mL)	1.7–8.6	13.74	26.7	1.63	5.57
Testosterone (ng/mL)	2.4–9.5	0.68	1.48	–	2.98
Oestradiol (pg/mL)	< 25	23.7	<20	192	–
Cortisol (µg/dL)	5–21	18.73	17.1		
ACTH (pg/mL)	5–46	67.2	37.1		
DHEAS (µg/dL)	80–560	267	129		
Androstenedione (ng/mL)	0.6–3.1	2.02	1.31		
Prolactin (ng/mL)	3.52–16.3	26	13.9		
Beta HCG (mIU/mL)	<5	NA	<1.2		
CEA (ng/mL)	<5	NA	1.01		
AFP (ng/mL)	<9	NA	<1		

#### Patient 2

This patient is the affected sibling of P1. The patient was referred for evaluation at the age of 12 years 7 months, due to gynecomastia and scrotal pain. His past medical history was unremarkable. At the presentation, his weight was 49.4 kg (+0.18 SDS) and his height was 154.8 cm (+0.01 SDS). Physical evaluation showed small, low-set, protruding ears, and bilateral gynecomastia of 5 cm with Tanner stage 4 appearance. Testicular volumes were 15 cm^3^ bilaterally and testes were painful at palpation. He had glanular hypospadias. Penile size was normal (9 × 2.5 cm). Pubic hair was at Tanner stage 3. Systemic examinations were normal. Karyotype was 46,XY. Adrenal function tests were normal, but serum gonadotropins were elevated suggesting hypergonadotropic hypogonadism; anti-Mullerian hormone concentration was high for his age and sex, serum inhibin A and B were undetectable, and tumour markers were negative (Table 1 and Supplementary Table 1, see section on supplementary materials given at the end of this article). Upon follow-up, he was treated with monthly intramuscular testosterone enanthate (50 mg/i.m.) for 4 months. His gonadotropins remained elevated and testosterone concentrations were below normal (Supplementary Table 1). He had two hospital admissions for bilateral scrotal pain with suspicion of testicular torsion. He was operated on due to varicocele on the left testis at 16 years and 2 months. At the last visit, at 17 years and 1 month, his weight was 69.3 kg (-0.01 SDS) and his height was 168.4 cm (−1.04 SDS). Systemic examinations were normal; he had bilateral gynecomastia of 5 cm with Tanner stage 4 appearance. Testicular volumes were 25 cm^3^ (right) and 30 cm^3^ (left) and testes were painful at palpation. Testicular ultrasound revealed some parenchymal heterogeneities, calcifications, varicocele, and hydrocele on repeated occasions (Supplementary Table 2). Sperm analysis, repeated three times at 18 years and 10 months, revealed azoospermia.

The father of the patient was 55 years old and had typical male external genitalia following normal pubertal onset and normal pubertal course. His gonadal function tests are seen in Table 1. Serum inhibin A and inhibin B concentrations were 306 and <1 pg/mL (N: 75–475 pg/mL and <2 pg/mL, respectively). Testicular ultrasound was normal (testis volumes: right, 38.6 cm^3^; left, 40 cm^3^).

The mother of the patient was 50 years old, with normal pubertal development and menarche at 12 years old. She still has regular menstrual bleeding and her gonadal function tests are seen in Table 1.

Two healthy sisters of the patients had regular menstrual cycles, which started at 12 and 13 years of age. One sister is 27 years of age, married and with two children.

### Molecular characterisations

WES (Patient 2 (P2)) and CES (P1) were performed. Identified variants were then excluded from consideration unless they met the following criteria: (i) variants with a MAF less than 0.01; (ii) variants in the coding sequence, splice variants, indels or duplications, and nonsynonymous changes; (iii) variants with a coverage of at least 20 reads; (iv) variants not recorded as benign in ClinVar database; (v) selecting pathogenic or disease-causing variants by all base conservation scores and functional prediction tools. Only one variant met all the above criteria: a homozygous variant (NM_002191.3:chr2:220437304_220437305delAG, c.208_209delAG, R70Gfs*3) in *INHA* (LOF Z-Score = 1.54, *n*  < 0.7). This variant was investigated for segregation with the disease in the parents by CES and the homozygous variant was also tested in P1 and P2 by Sanger sequencing (Fig. 1B, C and D). The variant was homozygous in patients and heterozygous in parents (Fig. 1B, C and D). We evaluated WES and CES data of P1 and P2 for all individual and shared homozygous, heterozygous and hemizygous variants of known genes causing testicular dysgenesis, impaired testosterone biosynthesis, disorders of sex development and primary gonadal failure, as well as pathogenic or likely pathogenic variants in the other genes. None of the individual or shared variants were associated with pathogenicity. Moreover, we have also evaluated all other *INHA* variants in CES and WES data of P1 and P2 for any other potentially pathogenic variants and found no other variants that can be associated with the phenotype. All sequence data are available on request.

The *INHA* variant identified in our patients was not found in 200 ethnically matched in-house Turkish exomes, the Turkish whole-exome database, nor in 100 in-house exomes from other Turkish 46,XY disorder/differences of sex developmentor hypogonadism patients. This variant was not seen in either GnomAD, ExAC, 1000 Genomes or 6500ESP.

## Discussion

This is the first report of male patients presenting with hypospadias, primary testicular failure, and infertility due to homozygous *INHA* mutations. Together, these data suggest significant roles for the inhibins in human sex development, steroidogenesis, and reproduction in 46,XY males.

The human *INHA* gene encodes a 366-amino acid inhibin subunit alpha protein, which is common to both inhibin A and B. Therefore, the homozygous 2-bp deletion mutation (c.208_209delAG, R70Gfs*3) identified in *INHA,*resulting in a frameshift in the coding sequence and a severely truncated inhibin subunit alpha protein, is predicted to cause significantly low serum inhibin A and B, as shown biochemically in P2. Although we were unable to measure serum activin levels, deficiency of inhibin subunit alpha protein could facilitate the formation of activin homodimers and enhance activin signalling in these patients. Hence, pituitary FSH secretion may increase the subsequent loss of negative feedback by inhibin and also potentially due to increased activin stimulation. High FSH induces constant stimulation of Sertoli cells, which could explain excessive AMH production, normal/high testicular volumes, and testicular pain observed in the patients. This contrasts with the general presentation of primary testicular failure, characterised by high FSH but low AMH and small testes. Male *Inha* knockout mice similarly exhibit testicular enlargement and grossly visible foci of haemorrhage by 5 weeks of age (6). However, these mice also develop testicular stromal tumours, which can contribute to increased testis size. Zebrafish lacking the inhibin a-subunit (*inha*^−^/^−^) also develop gonadal tumours in both sexes, similar to mice (9). Our patients did not develop tumours during their long follow-up period in adolescence and early adulthood. Nevertheless, the testicular USG of our patient revealed parenchymal heterogeneity and calcifications, which indicate close monitoring for the development of testicular tumours, considering the evidence from animal studies.

Testosterone deficiency was a major clinical feature of our patients. Knockdown of *Inha* expression impairs androgen biosynthesis by significant decreases in the expression of *Cyp17a1*, *Cyp11a1*, and *Nr5a1*, in mouse TM3 Leydig cell line (22). Similarly, knockdown of *Inha* expression in bovine ovarian theca cells resulted in suppression of androgen production subsequent to decreased *Cyp17a1* (10). It has been hypothesised that testosterone deficiency develops postnatally as a result of extensive somatic cell tumours causing Leydig cell regression and damage (6, 7, 8). However, both patients with *INHA* mutations had hypospadias and did not have testicular tumours, suggesting that androgen deficiency exists prenatally and impairs the hormone-dependent stage of external genitalia development in males. Postnatally, low testosterone promotes LH secretion, which could explain high LH concentrations in our patients, as an indirect effect of inhibin deficiency. This is in contrast to mice, in which inhibin has no effect on LH secretion (8). Additionally, adult rat Leydig cells and the MA-10 Leydig cell line provides support for the hypothesis that not only the lack of inhibin action but also unopposed activin signalling contribute to the decline in testosterone production when hypospermatogenesis is present (23). During human chorionic gonadotrophin (hCG) stimulation, activin A directly suppresses testosterone secretion but enhances progesterone secretion from rat Leydig cell primary cultures. Likewise, treatment of MA-10 cells with activin-A enhances cAMP-stimulated progesterone secretion and STAR expression (23).

Both patients with *INHA* mutations exhibited significant gynecomastia. Expression of ovarian aromatase (*cyp19a1a*) increases dramatically in the ovary of *inha*^−/−^zebrafish. Juvenile female mutants show signs of early follicle activation or precocious puberty onset (9). It is known that the testicular tumours in *Inha*^−/−^mice also produce excessive quantities of oestradiol (6, 7, 12). Although oestrogens were not high in these patients, gynecomastia could have developed as a result of disrupted testosterone/oestrogen balance due to low testosterone/oestrogen ratio or increased local aromatase activity.

Although the effects of inhibin on fertility have been reported in different species, sometimes with discrepancies, its functional importance in male reproduction remains largely unknown in humans. Knockout of the *Inha* gene in mice resulted in infertility in both females and males, mostly due to the formation of gonadal tumours and cachexia-related death (6). Deletion of *inha* in female zebrafish disrupted follicle development and maturation; however, in contrast to females, *inha* null male zebrafish showed normal spermatogenesis and fertility (9). In males, inhibin inhibits spermatogonial DNA synthesis (24) and reduces spermatogonia number (25), whereas activin stimulates spermatogonial proliferation *in vitro* (26). Inhibin synthesis by Sertoli cells fluctuates during the stages of spermatogenesis (27, 28). The binding of inhibin to different populations of germ cells (29) also changes during the various stages of spermatogenesis. These data support the critical intragonadal paracrine and/or autocrine role of inhibins, which mediate the interaction between Sertoli and germ cells. We have observed azoospermia and infertility in our patients with homozygous *INHA* mutations. Our results, therefore, emerge as the first human evidence of the physiological significance of inhibins in male reproduction. Some heterozygous missense variants in *INHA* including Ala257Thr, Ser92Asn, His175Gln, and Ala182Asp have been associated with POI, primary and secondary amenorrhoea in women (13, 30). Male and female mice heterozygous for the *Inha* deletion are normal and fertile (6). We identified no females homozygous for the *INHA* mutation in this family. However, the heterozygous mother and father of our patients had normal sex development, normal pubertal development and normal reproductive functions, and normal gonadotropin, AMH, and inhibin concentrations.

As testicular descent was normal in our patients, neither testosterone deficiency nor azoospermia could be explained by cryptorchidism.

As a potential limitation of our study, we could not perform activity studies of the *INHA* variant identified in our patients. Thus, it remains possible, although unlikely, that despite the severe truncation predicted due to the c.208_209delAG mutation of *INHA*, some biologic activity of the INHA protein is retained. Furthermore, inhibin and AMH concentrations could only be measured in P2. Nevertheless, available biochemical, clinical, and molecular findings support INHA deficiency in these two siblings – similar to the knockout animal models.

In conclusion, the clinical and molecular characteristics of these two patients suggest that homozygous *INHA* mutations cause decreased prenatal and postnatal testosterone production and infertility in males. Our results highlight the essential role of *INHA* and inhibins in human male sex development, testicular function, and reproduction.

## Supplementary Material

Supplemental Table 1. Gonadal function test results of patient 2 (P2) with INHA mutation in the follow-up.

## Declaration of interest

The authors declare that there is no conflict of interest that could be perceived as prejudicing the impartiality of this article.

## Funding

This work has been supported by the Medical Research Council of Marmara University (Project Grant SAG-A-120418-0152). Research in AG’s laboratory was funded by the UK Medical Research Council through core funding (MC_U142684167) at the Mammalian Genetics Unit, MRC Harwell Institute.

## Author contribution statement

E A A, M E, A G and T G designed the study. B G T, T S M, B S, S T and A B recruited and clinically characterised the patients. T G and M E conducted and analysed biochemical measurements. E A A performed and analysed the sequencing data. T G, A G, E A A and A B prepared the draft manuscript. All authors contributed to the discussion of results, and edited and approved the final manuscript.
