# Supplementary material for: Homozygosity for a novel INHA mutation in two male siblings with hypospadias, primary hypogonadism, and high-normal testicular volume
Source: Eur J Endocrinol. 2022 Mar 2;186(5):K25–31. doi: 10.1530/EJE-21-1230 (PMC9010807; doi:10.1530/EJE-21-1230)
Supplement: Supplemental Table 1. Gonadal function test results of patient 2 (P2) with INHA mutation in the follow-up. [file supplementary_table_1.pdf]

Supplemental Material

Supplemental Table 1. Gonadal function test results of patient 2 (P2) with *INHA* mutation in the follow-up.

**Age (yrs)**	**12.5**	**12.9**	**13.2**	**14.4**	**15.5**	**16.2**	**17.2**	**18.7**
**FSH (1.2-19.2 mIU/mL)**	91	74.7	105.5	90.1	60.6	59.9	58.8	48.3
**LH (1.7-8.6 mIU/mL)**	20.7	26.6	26.4	21.8	21.9	19.2	28.3	27.3
**Testosterone (2.4-9.5 ng/mL)**	1.48	1.77	1.42	1.63	2.14	1.73	1.56	1.33
**AMH (0.96-13.3 ng/mL)**					671*	331*	>24.5	192.3
**Inhibin B (75-475 pg/mL)**					<5			5.7
**Inhibin A (< 2 pg/mL)**								<1
**Testicular volume (cc)**	12	15	18	25	30		30	
**Testosterone treatment (mg/mo, IM)**				50	50			

* sample diluted 1/100

Serum LH and FSH levels were measured by Modular Analytics E 170 analyzer (Roche Diagnostics, Germany), which employs electrochemiluminescense immunoassay technology; the analytical sensitivities of these assays were <0.10 mIU/mL for both. The within-run and total coefficients of variations (CVs) of the FSH assay were less than 2.8% for concentrations ranging between 5.97-178 mIU/mL. For the LH assay, the within-run and total CVs were less than 1.2% for concentrations ranging between 6.15-164 mIU/mL. Serum anti-Müllerian hormone (AMH) levels were measured Cobas E 602 Module (Roche Diagnostics, Germany) with a measuring range of 0.01-23 ng/mL (0.071 - 164.2 pmol/L). The precision values were between 2.5-3.9% for a concentration range between 0.33-91.1 pmol/L (0.046-20.8 ng/mL). Serum inhibin A and B levels were measured employing chemiluminescense and enzyme-linked immunoassays from Beckman Coulter Inc. (High Wycombe, UK); respectively. The within run and between run total CVs for inhibin A were 4% and 3.97% with a measuring range of 1-1500 pg/mL, respectively. The within run and between run total CVs for inhibin B were ≤5.6% and ≤6.6 % with a measuring range of 2.91 - 1000 pg/mL, respectively.

Supplemental Table 2. Testicular ultrasound imaging results of patient 2 (P2) with *INHA* mutation.

	**12 yrs 6 mos**	**16 yrs 2 mos**	**18 yrs 6 mos**	**19 yrs 1 mos**
**Right testis size (mm)**	43x28x19	44x19		44x31x23
**Left testis size (mm)**	42x26x18	41x26		45x31x22
**Structure**	Punctate echogenities in left testis, venous dilatation of left pampiniform plexus (2.9 mm), heterogenous appearances of both testes with hypoechoic areas of undefinite borders, bilateral hydroceles in both hemiscrotums (right; 18x5 mm, left; 44x12 mm)	Varicocele on left testis (4 mm, reflux with Valsalva maneuver). 3-4 calcifications in left testis with maximum size of 9 mm	Venous dilatation of left pampiniform plexus (3.5 mm), varicocele on left testis (grade 3-4 mm, reflux with Valsalva maneuver), hydrocele in left hemiscrotum (3 cc), calcifications in left testis with maximum size of 9 mm	Hydrocele in left hemiscrotum, venous dilatation of left pampiniform plexus (right 1.5 mm, left 3.5 mm)
